# Transcriptome Profiling Analysis of Wolf Spider *Pardosa pseudoannulata* (Araneae: Lycosidae) after Cadmium Exposure

**DOI:** 10.3390/ijms17122033

**Published:** 2016-12-03

**Authors:** Chang-Chun Li, Yong Wang, Guo-Yuan Li, Yue-Li Yun, Yu-Jun Dai, Jian Chen, Yu Peng

**Affiliations:** 1 Hubei Collaborative Innovation Center for Green Transformation of Bio-Resources, College of Life Sciences, Hubei University, Wuhan 430062, China; lcc386@163.com (C.-C.L.); pearlyyl@yahoo.cn (Y.-L.Y.); panhui_hb@tom.com (J.C.); 2 Hubei Key Laboratory of Quality Control of Characteristic Fruits and Vegetables, Hubei Engineering University, Xiaogan 432000, China; wangyong@hbeu.edu.cn (Y.W.); lgy64662@sohu.com (G.-Y.L.); dyj5925@sina.com (Y.-J.D.)

**Keywords:** *P. pseudoannulata*, cadmium, transcriptome, RT-qPCR

## Abstract

*Pardosa pseudoannulata* is one of the most common wandering spiders in agricultural fields and a potentially good bioindicator for heavy metal contamination. However, little is known about the mechanisms by which spiders respond to heavy metals at the molecular level. In the present study, high-throughput transcriptome sequencing was employed to characterize the de novo transcriptome of the spiders and to identify differentially expressed genes (DEGs) after cadmium exposure. We obtained 60,489 assembled unigenes, 18,773 of which were annotated in the public databases. A total of 2939 and 2491 DEGs were detected between the libraries of two Cd-treated groups and the control. Functional enrichment analysis revealed that metabolism processes and digestive system function were predominately enriched in response to Cd stress. At the cellular and molecular levels, significantly enriched pathways in lysosomes and phagosomes as well as replication, recombination and repair demonstrated that oxidative damage resulted from Cd exposure. Based on the selected DEGs, certain critical genes involved in defence and detoxification were analysed. These results may elucidate the molecular mechanisms underlying spiders’ responses to heavy metal stress.

## 1. Introduction

Cadmium (Cd) is one of the most abundant, ubiquitous, toxic heavy metal elements in the environment [1]. In particular, many agricultural soils are significantly influenced by Cd derived from anthropogenic activities in many developing countries, such as China [2,3]. Cd can be absorbed by plant, and via food web accumulated in phytophagous insects and their predators [4,5], thus presenting a serious threat to ecosystem and human health. However, the diseases resulted from the long-term exposure to the sub-lethal concentration of heavy metals are difficult to be diagnosed in a timely manner, such as Itai-itai and Minamata diseases in Japan [6]. Therefore, how to monitor Cd pollution and assess its toxicological effects on organisms and environment are important environmental issues. 

Many studies have indicated that spiders have the ability to accumulate and withstand high concentrations of Cd [6,7]. Accumulated Cd can affect spiders’ biological traits, such as development and reproduction, biochemical and physiological processes [8,9,10]. Additionally, Cd can cause oxidative damage by stimulating the formation of free radicals and reactive oxygen species (ROS), resulting in oxidative stress [11], and can even display strong genotoxic effects and may cause DNA damage to spiders at low concentrations [12]. There is growing interest to use spiders as heavy metal indicators in ecotoxicological studies [6,13,14]. However, little is known about the mechanism of spider responses to Cd at the molecular level [9,11,15].

In recent years, the increasing use of high-throughput sequencing has provided us with an efficient and powerful platform to further study arachnids at molecular level [16,17,18]. Though the genes related to Cd response have been widely studied in many vertebrate and invertebrate [19,20,21], only a few genes of spider responding to heavy metals have been reported previously. The wolf spider *Pardosa pseudoannulata* (*P. pseudoannulata*) is one of the most common species of wandering spiders in agricultural fields in China, and acts as one of the most important natural enemies to reduce pest populations, such as rice plant hoppers and leafhoppers [22]. Our previous study indicated Cd can significantly affect the fitness-related traits and activities of antioxidative enzymes of *P. pseudoannulata* [10]. To further understand the biological basis of *P. pseudoannulata* response to Cd, there is a need to explore the transcriptomic biology of this spider species following Cd exposure.

The present study aimed to elucidate the molecular mechanisms and the critical genes involved in regulating spider responses to Cd stress. Accordingly, female adult spiders were exposed to 0, 0.2 and 2 mM CdCl_2_ solutions for seven days and their respective transcriptomes were compared for the first time, an abundance of differentially expressed Cd responsive genes were analysed. The molecular basis of the response to Cd stress was first comprehensively characterized in spider, and the resulting information would help in furthering our understanding of the toxicological mechanism of Cd, and using spiders as potential bioindicators of heavy metal contamination.

## 2. Results and Discussion

### 2.1. Sequence Analysis and De Novo Assembly

To study the mRNA expression dynamics of *P. pseudoannulata* exposed to different concentrations of CdCl_2_ solution, we constructed and sequenced mRNA-seq libraries from adult female spiders treated with lower (0.2 mM, TL), or higher (2 mM, TH) concentration of Cd, and distilled water as control (TC), respectively. After removing the low quality reads and trimming off the adapter sequences, 25,731,973 (TC), 25,542,476 (TL) and 28,212,795 (TH) high-quality, clean paired end sequencing reads with a total of 6,483,564,025, 6,435,605,510, and 7,108,718,203 bases were obtained, respectively. These high-quality reads were de novo assembled using the Trinity method. Reads that contain a certain length of overlap were first combined to form contigs, and then assembled contigs into transcripts, the longest transcript of each group was chosen as the unigene using TIGR gene indices clustering tool. A total of 60,489 unigenes were obtained with average length of 951 bp. The N50 values of these transcripts and unigenes were 1637 and 1433 bp, respectively. The unigenes with a length of 300 to 500 bp sequences represent the highest proportion, followed by 500–1000 bp sequences. A total of 16,257 (26.87%) unigenes were longer than 1000 bp, and 6390 (10.56%) were longer than 2000 bp (Table 1). This large dataset will contribute to the biochemistry analysis of this species as well as identification of important functional genes.

### 2.2. Annotation of Unigenes

A total of 18,773 of all (31.04%) unigenes were annotated by a BLAST search and alignment against the sequences in the nonredundant database, SwissProt database, Pfam database, COG database, GO database, KOG database and KEGG database, with an *E*-value threshold of le-5 (Table 2). Additionally, 41,716 (68.96%) were not annotated and may represent genes without detectable homologies. The number of annotated unigenes is lower than the results reported by other research groups for other invertebrate species [23,24], indicating that our knowledge of *P. pseudoannulata* genes is limited. Further research is needed to characterize these genes and explore their functions.

The *E*-value distribution of the top hits in the nr database showed that 55.81% of the sequences had strong homology (smaller than 1 × 10^−60^); 31.31% of the homology sequences ranged from 1 × 10^−60^ to 1 × 10^−15^; 20.99% had hits with similarity higher than 80% against the nr database; and 52.02% of the sequences with similarity higher than 60%. The species distribution showed that 76.63% of unigenes matched to four species, the unigenes had the highest homology to genes from *Stegodyphus mimosarum* (68.64%), followed by *Ixodes scapularis* (4.36%), *Zootermopsis nevadensis* (2.15%), and *Metaseiulus occidentalis* (1.49%) (Figure S1).

The unigenes of *P. pseudoannulata* were mapped to 26,522 GO terms and categorized into 54 subcategories, among which 5921 (22.32%) terms were assigned to 17 subcategories in cellular components, 8268 (31.17%) terms were assigned to 18 subcategories in molecular function, and 12,333 (46.5%) terms were assigned to 19 subcategories in biological processes (Figure S2). In addition, 6878 unigenes had significant matches in COG database. Among the 25 COG categories, the cluster for general function prediction only (27.74%) was the largest category, followed by replication, recombination and repair (11.02%); transcription (7.93%); signal transduction mechanisms (7.78%); posttranslational modification, protein turnover and chaperones (5.64%); amino acid transport and metabolism (5.41%); and translation, ribosomal structure and biogenesis (5.18%) (Figure S3).

A total of 11,737 unigenes participated in 254 KEGG pathways (Table S1), which were assigned to five categories, metabolism, genetic information processing, environmental information processing, cellular processes and organismal systems. Among them, the largest numbers of unigenes were assigned to signal transduction (2134 unigenes), endocrine system (1012 unigenes), nervous system (732 unigenes), immune system (616 unigenes), carbohydrate metabolism (494 unigenes) and transport and catabolism (492 unigenes). These results provided a valuable clue for investigating functional genes and specific biological processes in spider research.

### 2.3. Differential Expression Analysis

**Figure 1:**
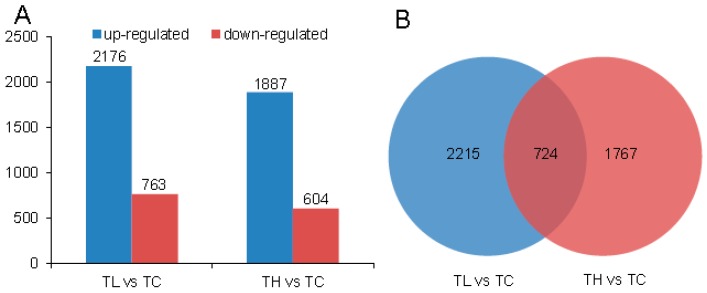
The differentially expressed genes (DEGs) numbers associated with 0.2 and 2 mM Cd exposure. TL: 0.2 mM CdCl_2_, TH: 2 mM CdCl_2_, TC: control. (**A**) The numbers of up- and down-regulated genes; (**B**) The numbers of DEGs in TL vs. TC and TH vs. TC.

To screen responsive genes, we calculated and compared expression levels among TL vs. TC and TH vs. TC groups, respectively, and thereby identified differentially expressed genes (DEGs) with an FDR < 0.01 and the absolute value of log_2_FC ≥ 1 taken as the selection criteria. As shown in Figure 1A, there were a total of 2939 DEGs between the TL and TC groups, in which 2176 and 763 unigenes were up-regulated and down-regulated, respectively. Between the TH and TC groups, there existed a total of 2491 DEGs, in which 1887 and 604 unigenes were up-regulated and down-regulated, respectively. These results clearly indicate that Cd treatment had a more relevant impact on gene up-regulation than on down-regulation. Furthermore, the number of DEGs observed after the 0.2 mM Cd treatment was slightly higher than that observed in 2 mM Cd-treated spiders. This is consistent with previous observations [25,26], which indicated that genes associated with Cd stress may have been caused by the lowering of the metabolic capacity of the living body by the strong toxicity of Cd content beyond a specific tolerance [27,28].

Through functional annotation, we separately obtained 1160 and 883 DEGs in TL vs. TC and TH vs. TC, respectively. Notably, 724 genes were commonly regulated by the two concentrations of Cd stress (Figure 1B). Significantly, these DEGs could be used to discover genes responsive to Cd stress in the spider, and thereby to identify some biomarkers for monitoring heavy metal pollution [29].

### 2.4. Functional Enrichment Analysis of DEGs

**Figure 2:**
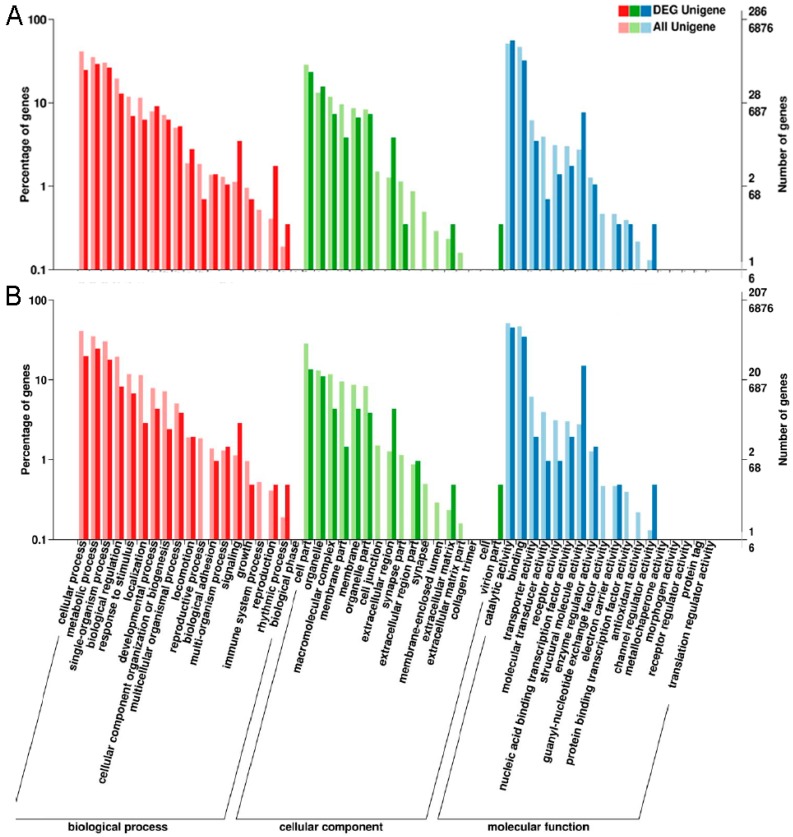
Gene ontology (GO) classification of differentially expressed genes (DEGs) in: TL vs. TC (**A**); and TH vs. TC (**B**). TL: 0.2 mM CdCl_2_, TH: 2 mM CdCl_2_, TC: control. The light histogram indicates all the annotated unigenes in each subcategory, the dark histogram indicates all the annotated DEGs in each subcategory. The left *y*-axis indicates the percentage of annotated unigenes or DEGs in that main category. The right *y*-axis indicates the number of annotated genes in each subcategory, the upper numbers correspond to the annotated DEGs, and the lower numbers correspond to the annotated unigenes.

To explore the pattern of transcriptome regulation of *P. pseudoannulata* following Cd exposure, all of the DEGs were performed on GO, COG functional annotation and pathway enrichment analysis. The DEGs between the groups (TL vs. TC and TH vs. TC) for the GO enrichment analysis were divided into three main clusters biological process (BP), cellular component (CC) and molecular function (MF), and thirty-eight, thirty-six and thirty-four subcategories under the three main GO categories were obtained, respectively. The predominant enriched subcategories were similar with 0.2 and 2 mM Cd exposure, “metabolic process” (GO: 0008152, part of BP), “cellular process” (GO: 0009987, part of BP), “catalytic activity” (GO: 0003824, part of MF) and “transporter activity” (GO: 0005215, part of MF) were both predominantly enriched in TL vs. TC and TH vs. TC (Figure 2). The enriched GO terms between the control and two Cd-treated groups in the three primary clusters were further analysed using the topGO software (enrichment significance KS < 0.05). The subcategories that topGO significantly enriched were consistent with GO predominantly enriched ones (Figure 2, Table S2). Moreover, signal transducer activity (GO:0004871, part of MF), molecular transducer activity (GO:0060089, part of MF) and cell surface receptor signaling pathway (GO:0007166, part of BP) were also significantly enriched GO terms in TL vs. TC and TH vs. TC, suggesting signal transduction systems of the spider were disturbed with Cd stress.

**Figure 3:**
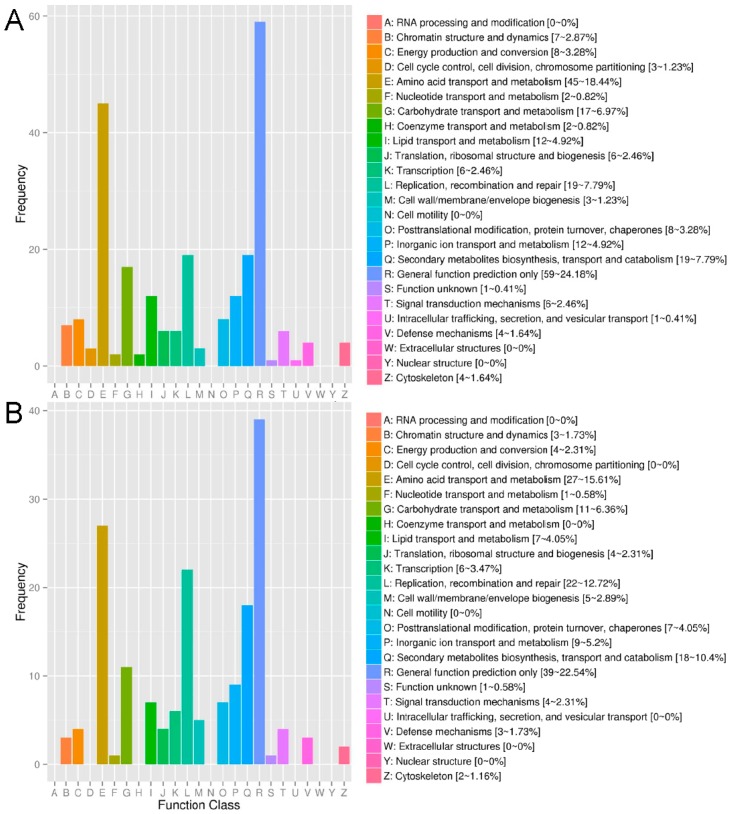
Clusters of orthologous (COG) classification of differentially expressed genes in: TL vs. TC (**A**); and TH vs. TC (**B**). TL: 0.2 mM CdCl_2_, TH: 2 mM CdCl_2_, TC: control.

The DEG annotation results in 26 COG classifications of TL vs. TC and TH vs. TC were shown in Figure 3. In TL vs. TC, the DEGs were mainly localized into the following four classifications: E (45, 18.44%), L (19, 7.79%), Q (19, 7.79%), and G (17, 6.97%). In TH vs. TC, E (27, 15.61%), L (22, 12.72%), Q (18, 10.4%), and G (11, 6.36%) were the four primary classifications. Moreover, more DEGs were located into “replication, recombination and repair” (L) in TH vs. TC than TL vs. TC, indicates that more serious DNA damage may be caused by higher concentration of Cd.

The KEGG Orthology-Based Annotation System was employed to search most statistically significantly enriched pathways for all the DEGs in TL vs. TC and TH vs. TC, and 194 and 120 differentially expressed pathways were identified, respectively. The predominantly enriched pathways were shown in Table 3. We then analysed the significance of the pathways using an enrichment factor and the Q-value, the results of the first 20 minimum Q-value pathways were displayed in Figure S4. Glycine, serine and threonine metabolism, fat digestion and absorption, drug metabolism-cytochrome P450 and Notch signalling pathway were both significantly enriched in TL vs. TC and TH vs. TC. It is worth noting that lysosome was significantly enriched pathway in TH vs. TC but not in TL vs. TC (Figure S4). One function of lysosome is known to degrade apoptotic cells, so the significantly enriched pathway of lysosome in TH vs. TC is probably due to more serious apoptosis resulting from higher concentration of Cd. Meanwhile, phagosome was predominantly enriched both in the groups TL vs. TC (8 DEGs) and TH vs. TC (5 DEGs), which indicated autophagy was probably induced by Cd [30].

When spiders are subjected to Cd stress, more energy is expended for defence (i.e., detoxifying the poisonous substance) [31,32,33]. Compared to the control groups, amino acid metabolism (COG, E), carbohydrate metabolism (COG, G), lipid metabolism (COG, I), energy metabolism (COG, C) and digestive system in COG and/or KEGG database were all shown to be significantly enriched, and most of the DEGs were significantly up-regulated, indicating the digestion and metabolism activities were actively induced to maintain the stability of their bodies when subjected to Cd stress.

The effects of heavy metal appear first at the molecular level and involve changes in polypeptide synthesis, the oxidation and denaturation of protein structures, DNA damage, intracellular respiration disorders and energy generation processes [11,12]. In extreme cases, high level of such damage suppresses metabolic processes and/or disintegrates cell organelles, leading to apoptosis [34,35,36]. When *P. pseudoannulata* were subjected to Cd stress, GO terms related to “metabolic process” (GO: 0008152), “catalytic activity” (GO: 0003824), “cell part” (GO: 0044464) and “organelle” (GO: 0044422), as well as DEGs involved in replication, recombination and repair (COG, L) and KEGG pathways in lysosomes and phagosomes were significantly enriched. These findings may indicate that oxidative damage in spiders at the cellular and molecular levels results from Cd exposure.

### 2.5. Analysis of Genes Related to Cd Detoxification

Transmembrane metal transporters are assumed to play key roles in heavy metal transport and detoxification [37,38,39]. Numerous studies focused on metal transporters in both plants and animals have been reported [19,23,39,40]. ATP-binding cassette (ABC) family transporters are important heavy metal transporters and interact with a wide range of chemicals including metals by pumping them across the cell membrane to maintain of cellular metal homeostasis [41,42]. In our study, the expression levels of ABC transporters were inhibited or induced. The expression of four of these genes was induced by Cd, one gene was suppressed in TL vs. TC groups and one gene in TH vs. TC groups was induced (Table S3). A relationship between ABC transporter expression and cadmium exposure has also been observed in other species [39,43,44].

Signal transduction is the main way for cells responding to heavy metal stress, when encounter an extra-cellular stimuli, the cell could activate a variety of specific stress-responsive signalling proteins to protect the cell [39,40]. Comparing the enriched DEGs of *P. pseudoannulata* in association with varying concentrations of Cd exposure, the “cell surface receptor signalling pathway” (part of BP, GO: 0007166) and “signal transducer activity” (part of MF, GO: 0004871) were significantly enriched. Various signalling pathways have been demonstrated to be associated with Cd exposure [19,45]. MAPK pathway is usually known to be activated by Cd via ROS generation, which is associated with signal transduction in response to oxidative stress, thus, also plays an essential role in eliminating oxidative damaged cells [46,47]. KEGG annotation indicated involvement of the following signalling pathways in all three libraries: Notch, MAPK, AMPK, Hedgehog, Ras and TNF. Many DEGs involved in the pathways were significantly up- or down-regulated with Cd exposure, confirming that the signal transduction of the spiders was disturbed by Cd contamination.

Heat shock proteins (Hsps) are critical factors during the process of environmental stress [19,48]. Genes encoding for Hsps, which play a vital role in the transport, folding, and assembly of proteins, are induced by various causal agents such as heavy metals [49,50,51]. In the present study, several forms of Hsps, including Hsp90, Hsp70, Hsp67, and Hsp60, and *hsp20* and *hsp70* genes were annotated in the unigene database. However, only the expression of Hsp70 and Hsp20 were significantly regulated (Table S3). This is consistent with the finding that *hsp20* and *hsp70* genes were substantially modulated in *Tigriopus japonicus* by heavy metals [50], and Hsp70 was significantly induced in *Cyprinus carpio* by Cd, indicating that both genes may be a good potential molecular biomarker for monitoring of heavy metal pollution [52].

Many studies have documented that Cd is often involved in oxidative stress resulting from the production of ROS [53,54]. Genes encoding detoxification enzymes played important role in preventing oxidative stresses and protecting organisms by the scavenging of ROS [55,56,57]. Glutathione metabolism played a pivotal role in protecting the organisms from heavy metal stress by quenching induced ROS, and GST is the most important enzyme of phase II detoxification and has a central role in defence against various environmental pollutants [58,59,60]. In the present study, several glutathione metabolism related enzymes were found based on the assembled transcriptome background, two transcripts encoding GST were detected over-expressed compared with the control in pathway of glutathione metabolism, indicating their important roles in the defence against Cd stress. Same results were recorded in digestive gland of *Mizuhopecten yessoensis* following Cd exposure [19]. Two other genes in glutathione metabolism pathways, including 5-oxoprolinase, gamma-glutamyltransferase were detected and all up-regulated in TL vs. TC or TH vs. TC. CYP450 is another critical detoxification enzyme considered to be a biomarker in many animals [19,29]. A total of 35 DEGs of CYP450 families were found in TL vs. TC and TH vs. TC groups and most of them presented up-regulate (Table S3), and these molecules may be used as biomarkers to assess the toxic effects of heavy metals on terrestrial invertebrates.

### 2.6. Validation of mRNA-Seq Data by RT-qPCR

To further evaluate the DEG library, nine transcripts with clear annotation were randomly selected for analysis by RT-qPCR. The RT-qPCR results displayed the same expression tendency as the DEG libraries (Table 4). The expression profiles of these nine genes were shown in Table 4, including cytochrome P450 4C1, Hsp 70, myb-related transcription factor, glutathione *S*-transferase, cytochrome P450 4c3, γ-Glutamyltransferase ywrD, ABC transporter and dimethylaniline monooxygenase.

## 3. Materials and Methods

### 3.1. Animal Materials and RNA Extraction

Subadult *P. pseudoannulata* specimens were collected from farm fields in Ma’anshan Forest Park, Wuhan (30°52′ N, 114°31′ E), Hubei Province, China, in April 2014. Spiders were kept individually in cylindrical glass tubes (diameter 2 cm, height 12 cm) with a layer of sponge (1.5 cm thick) moistened with distilled water on the bottom and fed in a chamber at 26 °C, relative humidity of 60%–80% under a light:dark cycle of 14:10 h (lights turned on at 08:00). Two days post-maturation, female adult spiders were exposed to 0.2 and 2 mM CdCl_2_ solution as their drinking water according to our previous study, and water for the control group [10]. We fed the spiders with *Drosophila melanogaster* and *Tendipes* sp., and replaced the moistened sponges every other day. Three biological replications were performed with each treatment containing at least six spiders. Seven days later, the treated spiders were immediately frozen in liquid nitrogen and stored at −70 °C refrigerator for RNA extraction.

The entire body (containing the carapace and abdomen) of each spider was used for RNA extraction, and then equal quality RNA of the three replicates of each group were mixed for mRNA-sequencing. Total RNAs were extracted using TRIzol Reagent (Huayueyang Biotech Co., Ltd., Beijing, China) following the manufacturer’s protocol and then treated with RNase-free DNase I (TaKaRa Biotech Co., Ltd., Dalian, China) to remove genomic DNA. RNA concentration, purity and integrity were determined through agarose gel electrophoresis and Agilent 2100 Bioanalyzer (Agilent Technologies, Inc., Santa Clara, CA, USA).

### 3.2. cDNA Library Construction, Sequencing and De Novo Assembly

The mRNA-seq libraries (TC, TL and TH) were constructed using the NEB Next Ultra RNA Library Prep Kit for Illumina (New England Biolabs, Inc., Ipswich, MA, USA), according to manufacturer’s protocol. In brief, magnetic beads with Oligo (dT) were used to isolate poly (A) mRNA from the high-quality total RNA samples. RNA fragmentation buffer was used to cut the mRNA into short fragments. Random hexamer primers and reverse transcriptase were used to synthesize the first-strand cDNA, and then the second-strand cDNA was synthesized by using buffer, dNTPs, RNaseH and DNA polymerase I. The cDNA was purified using a QiaQuick PCR extraction kit. The desired size of adaptor-ligated fragment with average inserts of 200 bp (150–250 bp) was selected by agarose gel electrophoresis. Polymerase chain reaction (PCR) was performed to selectively enrich and amplify the selected cDNA fragments to construct cDNA libraries. The resultant libraries were tested for quality conformance and sequenced using the Illumina HiSeq™ 2500 (Biomarker Technologies Co., Ltd., Beijing, China) to generate 125 bp paired-end read lengths.

Subsequently, the obtained raw reads were processed to get clean reads by removing adaptor sequences and low quality bases. Clean reads obtained were randomly clipped into short fragments (K-mers) by applying Trinity software (v2.0.2) [61]. The K-mers with a certain length of overlap were combined to form longer fragments, contigs, and the overlap between these contigs was utilized to build graph components. Then, the clean reads were mapped back to obtain transcripts using the method of the De Bruijn graph. Finally, the unique assembled transcripts were further subjected to the process of sequences-splicing redundancy removal using TIGR gene indices clustering tools [62] to acquire non-redundant transcripts called unigenes.

### 3.3. Unigene Functional Annotation

All the unigenes were searched against NCBI non-redundant nucleotide collection (Nr/nt), Swiss-Prot, Gene Ontology (GO), Clusters of Orthologous Groups (COG), and Kyoto Encyclopedia of Genes and Genomes (KEGG) protein information databases using BLASTx to obtain annotation information. The parameter *E*-value ≤ 1 × 10^−5^ of BLASTx was taken as a threshold of significant similarity.

### 3.4. Differential Dene Expression and Functional Annotation Analyses

For gene expression analysis, all clean reads were aligned to the unigene library using Bowtie software [63], and the obtained results were further used to estimate expression level using RSEM software [64]. Fragments Per Kilobase of transcript per Million (FPKM) for each unigene from the three separate libraries was calculated to show the expression quantity of the gene in the sample [65]. Differentially expressed genes (DEGs) between the groups (TL vs. TC and TH vs. TC) were analysed using EBSeq software respectively [66]. Benjamini–Hochberg method was used to correct the significance of the *p*-value [67]. In this study, we used corrected *p*-values that called false discovery rate (FDR) <0.01 and the absolute value of log_2_ fold change (FC) ≥1 as the threshold to judge the significant difference in gene expression.

All DEGs between the groups (TL vs. TC and TH vs. TC) were carried on GO, COG functional annotation and KEGG pathway enrichment analysis. The topGO software [68] was used for GO enrichment analysis by the “elim” method with a minimum node size of 6. DEGs in the KEGG pathway enrichment degree were statistically analysed by the enrichment factor and corrected *p*-value (*q*-value) using the following formula: enrichment factor = (the number of DEGs in pathway/the number of DEGs)/(the number of all unigenes in pathway/the number of all unigenes in KEGG). The larger the enrichment factor is, the more significant of the enrichment level for DEGs, and the smaller the log value of *q*-value is, the more reliable of the enrichment significant for DEGs.

### 3.5. Validation of mRNA-Seq Data

Total RNA of the spiders was extracted by using TRIzol method (Taraka, Japan) from the previous samples. One microgram of total RNA was used in reverse transcription in total volume of 20 μL in the presence of 6-mer random primer and oligo primer according to the protocol of Taraka. Quantitative real-time PCR was performed on a Viia™ 7 Real-Time PCR System (ABI, Waltham, MA, USA) platform using the SYBR Premix Ex Taq™ II (Tli RNaseH Plus, ROX plus), (TaKaRa, Japan) following the manufacturer’s instructions. We selected nine DEGs (c59601.graph_c0, c82407.graph_c1, c91015.graph_c0, c92582.graph_c0, c98395.graph_c0, c98430.graph_c0, c101656.graph_c1, c91520.graph_c0 and c87886.graph_c0) for RT-qPCR validation (Table S4). The primer sets were designed using Premier 5.0 software. The amplification was achieved by the following PCR program of first denaturation 95 °C for 30 s, followed by 40 cycles of 5 s at 95 °C, 30 s at 55 °C and 30 s at 72 °C, then a melting curve analysis was conducted from 60 to 95 °C. All samples were tested in triplicate, and the experiments were performed on three biological replicates. The relative expression levels of the selected transcripts normalized to the internal control gene (*β-actin*) were calculated using the 2^−ΔΔ*C*t^ method.

## 4. Conclusions

In summary, the present study firstly represents a comprehensive transcriptome characterization of *P. pseudoannulata* following Cd exposure through high depth sequencing. A total of 60,489 assembled unigenes were obtained, 18,773 of which were annotated in the public databases. A total of 2939 and 2491 DEGs were detected between the two Cd-treated and control libraries in the spider. Metabolism processes and digestive system were predominately enriched with Cd exposure. Significantly enriched pathways in lysosome, phagosome, and replication, recombination and repair demonstrated the oxidative damage resulted from Cd at cellular and molecular levels. Based on the DEGs analysis, multiple candidate genes involved in defence and detoxification were successfully identified in response to Cd stress. These data provide potential molecular targets in *P. pseudoannulata* for functional studies of genes responding to Cd stress and may serve as a valuable reference for identifying biomarkers in Cd pollution monitoring.

## Figures and Tables

**Table 1:** The length distribution of transcripts and unigenes.

Length Range (bp)	Transcript	Unigene
0–300	11,907 (10.02%)	0 (0%)
300–500	40,345 (33.95%)	27,245 (45.04%)
500–1000	31,410 (26.43%)	16,985 (28.08%)
1000–2000	20,515 (17.26%)	9867 (16.31%)
2000+	14,674 (12.35%)	6390 (10.56% )
Total number	118,853	60,489
Total length	119,999,996	57,521,612
N50 length	1637	1433
Mean length	1009.65	950.94

**Table 2:** Functional annotation of the *P. pseudoannulata* transcriptome.

Databases Used for Annotation	Annotated Number	300 ≤ Length < 1000	Length ≥ 1000
COG	5131 (8.48%)	1312	3819
GO	6876 (11.37%)	2310	4566
KEGG	8297 (13.72%)	2484	5813
KOG	11,804 (19.51%)	3694	8110
Pfam	13,550 (22.40%)	4204	9346
Swissprot	10,216 (16.89%)	3164	7052
nr	18,477 (30.55%)	7477	11,000
All	18,773 (31.04%)	7709	11,064

**Table 3:** The predominantly enriched pathways in the groups TL vs. TC and TH vs. TC.

TL vs. TC	TH vs. TC
Glycine, serine and threonine metabolism (11 DEGs)	Lysosome (7 DEGs)
Lysosome (10 DEGs)	Fat digestion and absorption (7 DEGs)
Fat digestion and absorption (10 DEGs)	Fatty acid elongation (5 DEGs)
Protein processing in endoplasmic reticulum (9 DEGs)	Protein processing in endoplasmic reticulum (5 DEGs)
Phagosome (8 DEGs)	Phagosome (5 DEGs)
Carbon metabolism (8 DEGs)	Notch signalling pathway (4 DEGs)
Pyruvate metabolism (7 DEGs)	Peroxisome (4 DEGs)
Drug metabolism-cytochrome P450 (7 DEGs)	Glycine, serine and threonine metabolism (4 DEGs)
Oxidative phosphorylation (6 DEGs)	Valine, leucine and isoleucine degradation (4 DEGs)
Notch signalling pathway (6 DEGs)	Glutathione metabolism (3 DEGs)
Valine, leucine and isoleucine degradation (6 DEGs)	Drug metabolism-cytochrome P450 (3 DEGs)

**Table 4:** Validation of the RNA-Seq expression profiles of selected DEGs by RT-qPCR.

Transcript ID	Brief Description	DEG Library	Fold by RNA-Seq	Fold by qPCR
c59601.graph_c0	Cytochrome P450 4C1	TL vs. TC	7.78	21.55
c82407.graph_c1	Heat shock protein 70	TL vs. TC	−2.53	0.26
c91015.graph_c0	Myb-related transcription factor	TH vs. TC	7.39	4.65
c92582.graph_c0	Glutathione *S*-transferase	TL vs. TC	2.80	4.41
c98395.graph_c0	Cytochrome P450 4c3	TL vs. TC	9.06	42.27
		TH vs. TC	8.47	15.51
c98430.graph_c0	gamma-Glutamyltransferase	TL vs. TC	2.56	2.93
		TH vs. TC	2.27	2.24
c101656.graph_c1	ABC transporter	TL vs. TC	2.24	4.96
c91520.graph_c0	Glutathione *S*-transferase	TL vs. TC	2.62	10.44
		TH vs. TC	3.24	21.13
c87886.graph_c0	Dimethylaniline monooxygenase	TL vs. TC	7.60	315.25

## Supplementary Materials

Supplementary materials can be found at www.mdpi.com/1422-0067/17/12/2033/s1.
