# Supplementary material for: Transcriptome Profiling Analysis of Wolf Spider Pardosa pseudoannulata (Araneae: Lycosidae) after Cadmium Exposure
Source: Int J Mol Sci. 2016 Dec 3;17(12):2033. doi: 10.3390/ijms17122033 (PMC5187833; doi:10.3390/ijms17122033)
Supplement: Supplementary file 1 [file ijms-17-02033-s001.zip › ijms-157326-Supplementary Materials/ijms-157326-supplementary.pdf]

Supplementary Material: Transcriptome Profiling Analysis of Wolf Spider *Pardosa pseudoannulata* (Araneae: Lycosidae) after Cadmium Exposure

Chang-Chun Li, Yong Wang, Guo-Yuan Li, Yue-Li Yun, Yu-Jun Dai, Jian Chen and Yu Peng

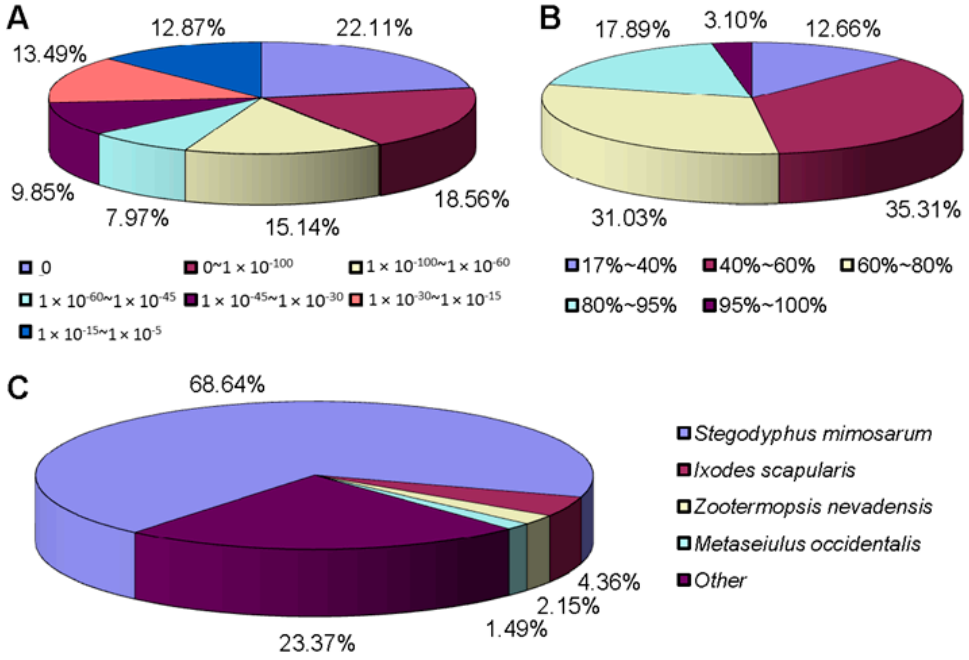

Figure S1. Characteristics of similarity search of unigenes against nr databases. (**A**) *E*-value distribution; (**B**) Similarity distribution; (**C**) Species distribution.

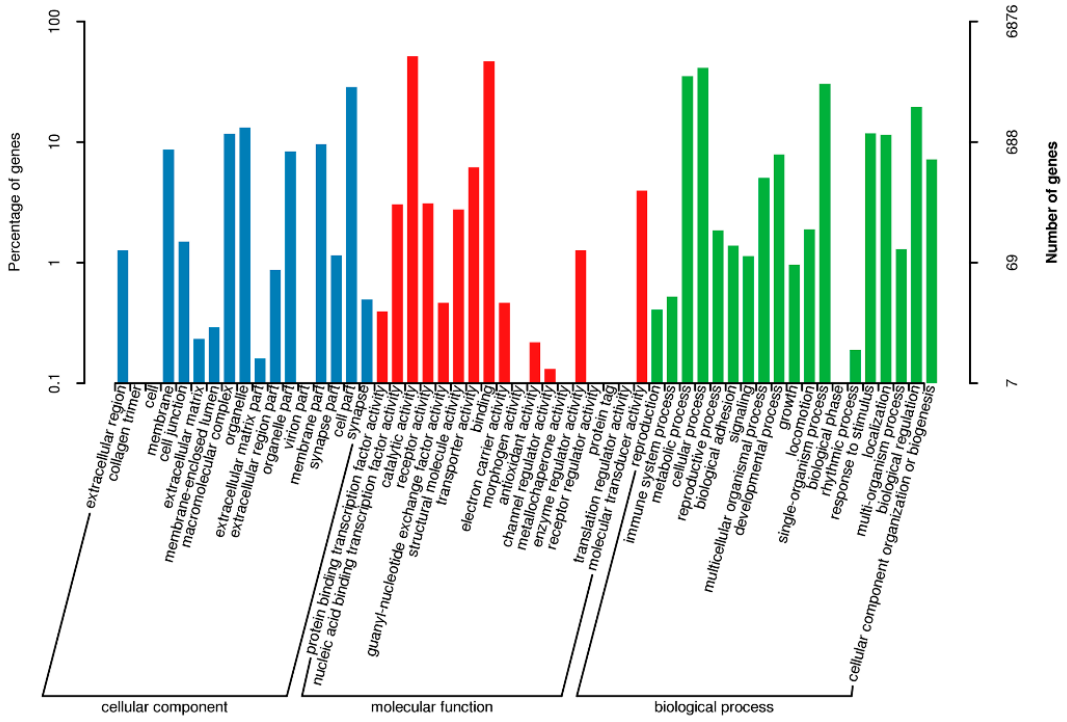

Figure S2. Gene ontology (GO) functional classification of unigenes. Unigenes were annotated for subcategories in one of the three categories: biological processes, cellular components and molecular functions.

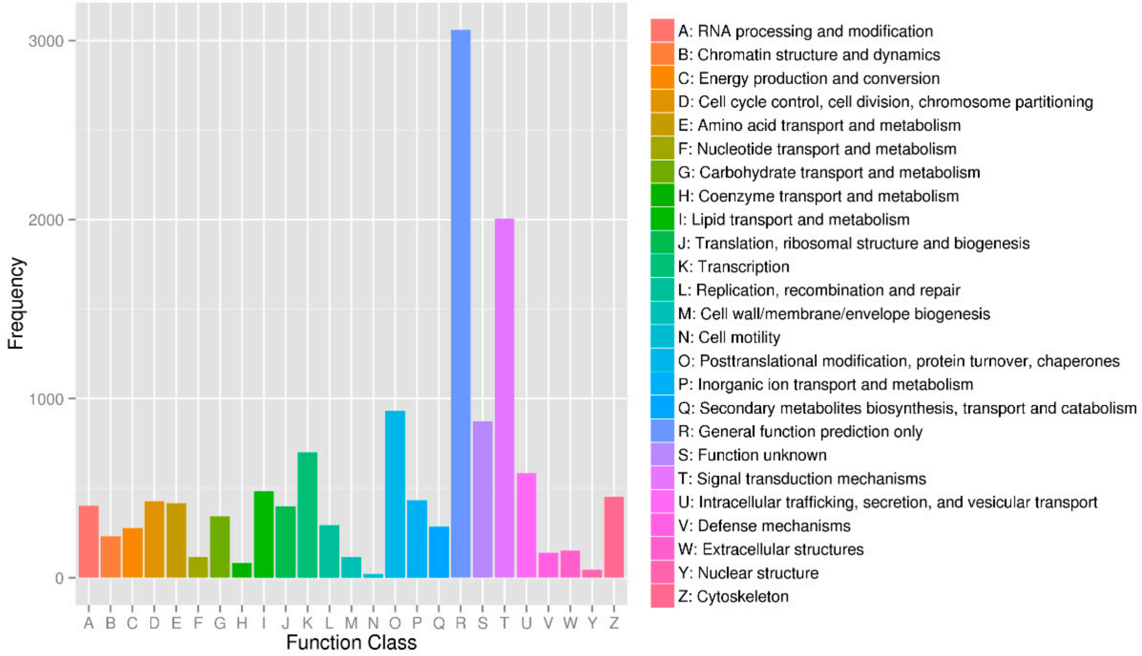

Figure S3. Clusters of orthologous (COG) classification of unigenes. A total of 6,685 unigenes were grouped into 25 COG classifications. The *y*-axis indicates the number of genes in a specific function cluster. The legend shows the 25 function categories.

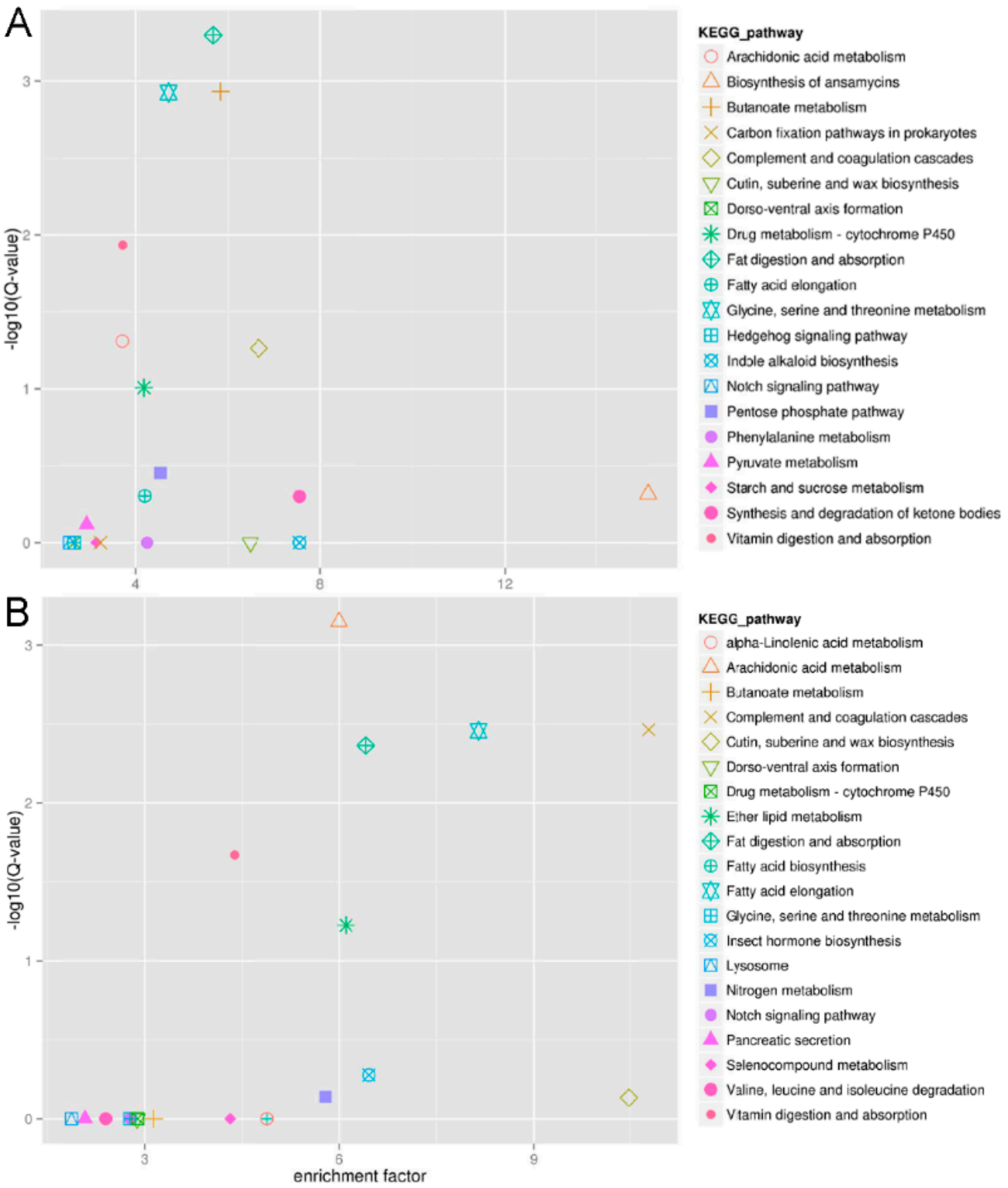

Figure S4. Kyoto encyclopedia of genes and genomes (KEGG) pathways enrichment analysis of differentially expressed genes in TL vs. TC (**A**) and TH vs. TC (**B**). TL: 0.2 mM $CdCl_2$, TH: 2 mM $CdCl_2$, TC: control.
